# Interface-controlled broadband photodetection in Si_3_N_4_/n-Si hybrid devices

**DOI:** 10.1038/s41598-026-57951-y

**Published:** 2026-06-16

**Authors:** Theodore Azemtsop Manfo, Dilber Esra Yıldız, Cengiz Bağcı, Ali Akbar Hussaini, Murat Yıldırım

**Affiliations:** 1 Department of Electrical Engineering and Energy Technology, School of Technology and Innovations, University of Vaasa, Wolffintie 32, 65200 Vaasa, Finland; 2 Department of Physics, Faculty of Engineering and Natural Sciences, Hitit University, 19030 Corum, Turkey; 3 Department of Metallurgical and Materials Engineering, Faculty of Engineering and Natural Sciences, Hitit University, 19030 Corum, Turkey; 4 Department of Biotechnology, Faculty of Science, Selcuk University, 42130 Konya, Turkey

**Keywords:** Si_3_N_4_ interfacial layer, n-Si photodiodes, Hybrid photodetectors, Interface engineering, Responsivity, Specific detectivity, Materials science, Optics and photonics, Physics

## Abstract

In this study, Si_3_N_4_/n-Si hybrid photodiodes with distinct interfacial layer configurations (Si_3_N_4_-1, Si_3_N_4_-2, and Si_3_N_4_-3) were constructed and comprehensively examined to evaluate the influence of interface engineering on their optical performance. Measurements of current–voltage (I–V) under dark and illuminated settings show that the ideality factor (n) declines with increasing illumination intensity. However, the barrier height (Φ_B_) shows structure-dependent variation, indicating enhanced carrier transport and interface modulation. To assess device performance under varying light intensities, responsivity (R), photosensitivity (K), noise specific detectivity (D*) and equivalent power (NEP) were determined. The Si_3_N_4_-3/n-Si structure had the highest photosensitivity, while the Si_3_N_4_-2/n-Si device showed superior overall performance with enhanced responsivity, higher detectivity, and lower NEP, indicating an optimal balance between photocarrier generation and transport. Spectral investigation confirmed a broadband response with higher external quantum efficiency (EQE) in the UV–Vis-NIR region. The linear relationship between log(I_ph_) and log(P) features indicates a dominating photoconduction mechanism impacted by trap states within the mobility gap. The results show that precisely controlling the Si_3_N_4_ interfacial layer improves photodiode performance by minimizing recombination losses and improving carrier dynamics. These findings show that Si_3_N_4_-based interface engineering is a promising method for producing high-performance, broadband silicon photodetectors for advanced optoelectronic applications.

**Supplementary Information:**

The online version contains supplementary material available at https://doi.org/10.1038/s41598-026-57951-y.

## Introduction

Semiconductor devices constitute the foundation of modern optoelectronics, serving as the core elements in technologies ranging from photodiodes and photodetectors to photosensors, optoelectronic transistors, and photovoltaic cells^1^. Tunable properties are essential for applications in nano/microelectronics and optoelectronics^1^. Photodiodes typically consist of layered structures including electrodes for charge collection, an active layer that generates electron–hole pairs, and a blocking layer to reduce leakage current. They are usually operated under reverse bias to enhance carrier collection efficiency^2^. Organic photodiodes (OPDs) have gained attention due to their flexibility, low cost, and lightweight nature^3–5^. Meanwhile, metal–semiconductor (MS) interfaces, or Schottky diodes, are widely studied for their simple fabrication, fast response, and use in optoelectronic and sensing devices^6,7^. Unlike conventional p–n junctions, Schottky diodes rely on the metal–semiconductor interface for rectification, enabling rapid charge transport. With increasing demand for flexible and scalable technologies, integrating organic semiconductors into MS structures has become a promising approach for developing hybrid photodetectors with tunable and enhanced optoelectronic performance^8,9^.

Photodetectors represent a fundamental class of optoelectronic components that transduce incident photons into measurable electrical signals, thereby underpinning diverse applications including optical communication, environmental monitoring, imaging systems, and advanced sensing platforms. Among various material systems, silicon (Si)‑based photodetectors have recently garnered significant research interest owing to their inherent compatibility with complementary metal–oxide–semiconductor (CMOS) processes, cost‑effective fabrication routes, and superior electronic characteristics. However, conventional Si photodetectors often suffer from limited spectral response, high recombination losses, and reduced sensitivity, particularly in broadband detection applications^10,11^. Significant advances have been made by integrating dielectric layers such as silicon nitride (Si_3_N_4_), Silicon carbide (SiC), or silicon oxide (SiO_2_)^12,13^. The addition of these layers not only improved the optical and electrical properties but also broadened the range of wavelengths that could be absorbed, including the ultraviolet (UV). This invention has opened new possibilities for the advancement of optoelectronic devices^14,15^.

SiC, Si_3_N_4_, and SiO_2_ have emerged as viable options for dielectric matrices. SiO_2_, due to its greater bandgap than Si_3_N_4_, produces higher barriers, which have a significant impact on transport characteristics^12,13,16^. The Si_3_N_4_/Si interface has generated great interest in the scientific community because of its higher carrier tunneling probability and decreased barrier height, making it a highly promising option for Si-based electronics. To address these restrictions, extensive research has been conducted on heterostructure and interface engineering approaches, such as the integration of dielectric and wide-bandgap materials with silicon. Si_3_N_4_ is a potential interfacial material with great optical transparency, chemical stability, a wide bandgap, and good passivation capabilities. Si_3_N_4_ is very well suited for improving carrier transport and lowering interface trap densities in silicon-based photodiodes because of these characteristics^17^. Si_3_N_4_ is compatible with silicon photonics platforms, making it a promising material for next-generation integrated optoelectronic devices^18^.

Recent research shows that using Si_3_N_4_ as an interfacial layer in metal-insulator-semiconductor (MIS) or Schottky-type photodiodes can significantly increase device performance. A thin Si_3_N_4_ layer enhances energy band alignment and carrier separation efficiency, minimizing surface recombination, dark current, and boosting responsiveness and detectability^17^. Furthermore, hybrid and waveguide-integrated photodetectors based on silicon platforms have demonstrated significant improvements in broadband detection and high-speed operation, highlighting the importance of interface engineering in modern photodetector design^11,18^.

Furthermore, recent research on silicon-compatible and nanostructured photodetectors has shown that interfacial features, including defect density, band alignment, and carrier transport pathways, have a significant impact on device performance. Optimized interface layers can increase photocarrier production and collection efficiency while reducing recombination losses^9,17^. Despite these developments, attaining the best balance of photosensitivity, responsivity, noise performance, and spectrum response remains a significant issue. The thickness and quality of the interfacial layer must be carefully managed, since excessive thickness can introduce additional series resistance and impede efficient carrier transmission.

The current study seeks to analyze the impact of Si_3_N_4_ interfacial layer engineering on the performance of Si-based hybrid photodiodes. Three device configurations, Si_3_N_4_-1/n-Si, Si_3_N_4_-2/n-Si, and Si_3_N_4_-3/n-Si, are fabricated and analyzed to evaluate the effect of varying interfacial layer properties on key performance parameters, including K, R, NEP, D*, and EQE. This work aims to determine the optimal interfacial arrangement for carrier production, transport efficiency, and recombination kinetics by evaluating different designs. The findings are expected to provide important insights into interface engineering strategies for developing high-performance broadband photodetectors that are compatible with silicon-based optoelectronic systems.

## Experimental

### Characterization of synthesized of Si_3_N_4_ powders

Three different Si_3_N_4_ powders were used in this study for comparative assessment. The commercial powder (Si_3_N_4_-1) was obtained from the industrial supplier (≤ 10 μm, Sigma-Aldrich, Germany). The other two powders, Si_3_N_4_-2 and Si_3_N_4_-3, were synthesized from as-received sepiolite and enriched-sepiolite, respectively, following the procedure reported by Bagci et al.^20^. Sepiolite used as a silica source was obtained from Turktaciri location in south of Ankara. The as-received sepiolite is a hydrated magnesium silicate clay mineral with the formula of Mg_8_Si_12_O_30_(OH)_4_(OH_2_)_4_ 8H_2_O and has XRD-detected 10% dolomite (CaMg(CO_3_)_2_). The dolomite was removed by treating it in an HCl solution, and thus the sepiolite was enriched to improve the purity of Si_3_N_4_ that will be synthesized. The synthesis involved the reduction of silica of both as-received and enriched sepiolite to silicon separately with carbon black and simultaneous nitriding in a dynamic nitrogen atmosphere by the carbothermal reduction and nitridation method.

### Fabrication of Al/Si_3_N_4_/n-Si/Al photodetector devices

**Fig. 1:**
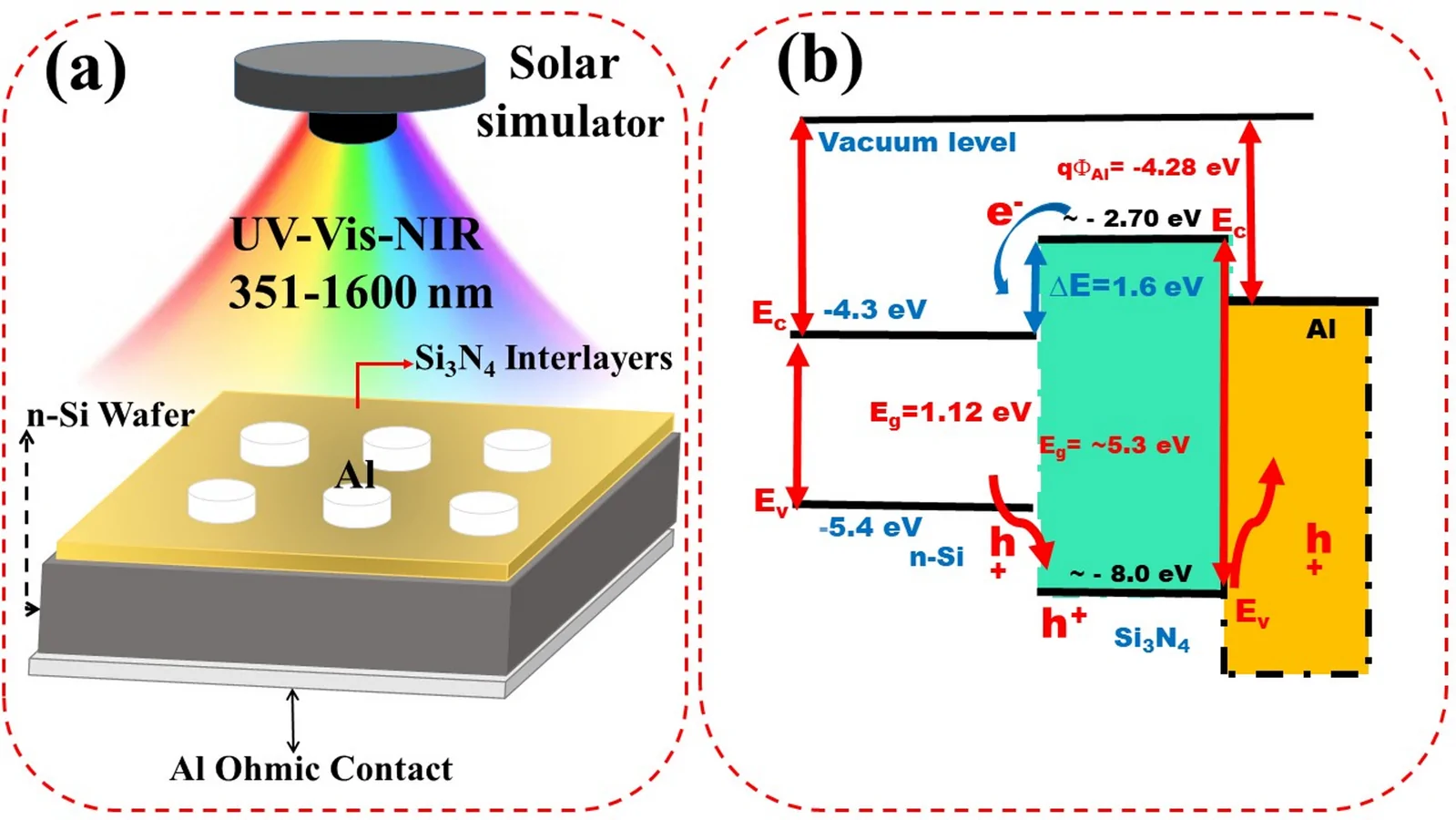
(**a**) A schematic representation and (**b**) The energy band diagram of the Al/Si_3_N_4_/n-Si devices.

Phosphorus-doped silicon wafers were obtained from Wafer World and cleaned using the RCA cleaning procedure as described in^21^. The back (ohmic) contacts of the n-Si wafers were deposited by physical vapor deposition (PVD) under a vacuum of 7 × 10^− 6^ Torr, with a thickness of 120 nm. A subsequent thermal treatment (450 °C) was applied to form an ohmic Al contact. Si_3_N_4_ solutions were prepared, and 50 µL was drop-cast onto the front surface of the n-Si wafer. The samples were then spin-coated at 2000 rpm for 40 s to achieve uniform coverage. The coated wafers were heated at 100 °C to remove residual solvent. Metallic contacts were subsequently deposited onto the Si_3_N_4_ interlayer using the same PVD conditions (7 × 10^− 6^ Torr, 120 nm thickness) as used for the front (Schottky) contact. The active surface area of the device was 7.85 × 10^− 3^ cm^2^. A schematic representation of the device is shown in Fig. 1a. The energy band diagram illustrates the electronic alignment and carrier transport pathways within the Al/Si_3_N_4_/n-Si heterostructure in Fig. 1b. The work function of Al (φ_m_ ≈ −4.28 eV) is shown relative to the conduction and valence band edges of n-Si (E_c_ = − 4.3 eV, E_v_ = − 5.4 eV, E_g_ = 1.12 eV) and the wide bandgap dielectric Si_3_N_4_ (E_g_ ≈ 5.3 eV, E_v_ ≈ − 8.0 eV)^17^. The interlayer introduces a barrier offset (ΔE ≈ 1.6 eV) between conduction bands, thereby modulating electron injection and suppressing leakage currents. Arrows indicate the photoexcited electron (e⁻) and hole (h⁺) transport across the interfaces under solar illumination. This band alignment highlights the role of Si_3_N_4_ in stabilizing the junction, enhancing charge separation, and supporting broadband photoresponse in the UV–Vis–NIR spectral range.

### Characterization

Further morphology of the synthesized powders was characterized using scanning electron microscopy (SEM Supra 50VP, Zeiss, Germany), and their local chemical compositions were determined by Energy Dispersive X-ray Spectroscopy (EDS). Measurements were carried out on selected regions to obtain representative compositional data. The electrical and optoelectrical measurements of the Al/Si_3_N_4_/n-Si/Al devices were carried out using an FY7000 solar simulator and a Keithley 2400 source meter. The measurements were performed under simulated solar light intensities of 20, 40, 60, 80, and 100 mW/cm^2^. Additionally, wavelength-dependent measurements were conducted using a set of visible hard-coated bandpass filters. Current–transient measurements were recorded at 0 V over a wavelength range of 351 to 1600 nm. According to pyranometer readings, all LEDs used during testing provided nearly uniform illumination power density. The optical power intensity for each selected wavelength was maintained at approximately 20 mW/cm^2^.

## Results and discussion

**Fig. 2:**
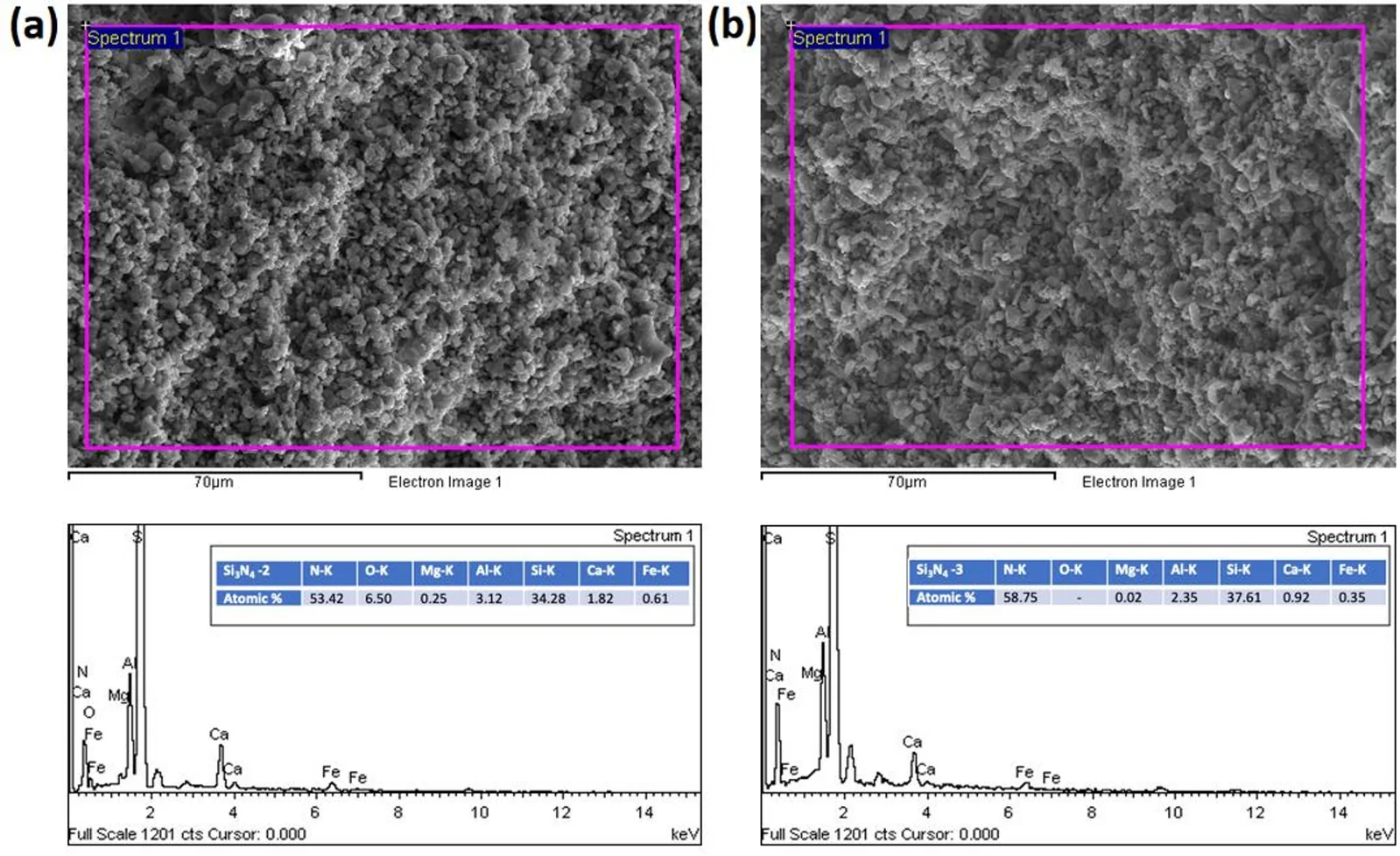
SEM-EDS analysis of compacted Si_3_N_4_ powders (Si_3_N_4_-2 and Si_3_N_4_-3) synthesized from sepiolite and correlated with Si_3_N_4_/n-Si photodiode performance, highlighting how precursor purity and interfacial engineering enable high efficiency while leveraging a sustainable approach.

In this work, three Si_3_N_4_-based hybrid photodiodes were fabricated and analyzed. SEM observations revealed that both synthesized powders (Si_3_N_4_-2 and Si_3_N_4_-3) consist of highly agglomerated particles with irregular and porous morphologies (Fig. 2a,b). These agglomerates are consisted of fine primary grains in the nano- to submicron scale. Such morphology is typical for Si_3_N_4_ powders produced via carbothermal reduction and nitridation of clay-based silica sources, where the original fibrous structure of sepiolite collapses during thermal processing.

A difference between the two synthesized powders was observed. Si_3_N_4_-3, synthesized from enriched sepiolite, revealed a more homogeneous and relatively refined microstructure. Yet, Si_3_N_4_-2 showed a broader particle size distribution and more pronounced heterogeneity. This difference is attributed to the enrichment of the precursor, which enhances reaction uniformity and reduces the presence of non-siliceous phases.

EDS analyses confirmed that Si and N are the dominant elements in both synthesized powders, consistent with Si_3_N_4_ formation. However, Si_3_N_4_-2 also contained detectable amounts of O, Mg, Al, Ca, and Fe. These impurities originate from the natural composition of as-received sepiolite and indicate incomplete removal of other mineral phases. The presence of oxygen suggests either residual silica (SiO_2_) or surface oxidation of Si_3_N_4_ particles. In contrast, Si_3_N_4_-3 showed a higher relative Si content and significantly reduced impurity levels. Oxygen was below the reliable detection limit in the EDS spectrum, indicating either very low oxide content or the presence of an ultra-thin surface oxide layer. This result suggests a more complete nitridation reaction and improved chemical purity due to the beneficiation process.

The phase composition of the synthesized powders was not re-examined in this study; instead, previously reported XRD results^20^. According to their findings, both powders predominantly consist of Si_3_N_4_, after nitridation. Minor secondary phases, such as residual silica and impurity-related oxides, were reported particularly for the Si_3_N_4_-2. These literature findings are consistent with the present SEM–EDS results. The higher impurity content and detectable oxygen in Si_3_N_4_-2 correlate with XRD patterns of Si_3_N_4_ obtained from as-received sepiolite. In contrast, the improved purity of Si_3_N_4_-3 is in agreement with the reduction of amorphous region.

Compared to the synthesized powders, the commercial Si_3_N_4_-1 is expected to have higher purity, a controlled particle size distribution, and lower oxygen content due to optimized industrial processing. These characteristics generally result in improved sintering performance and more predictable material properties.

**Fig. 3:**
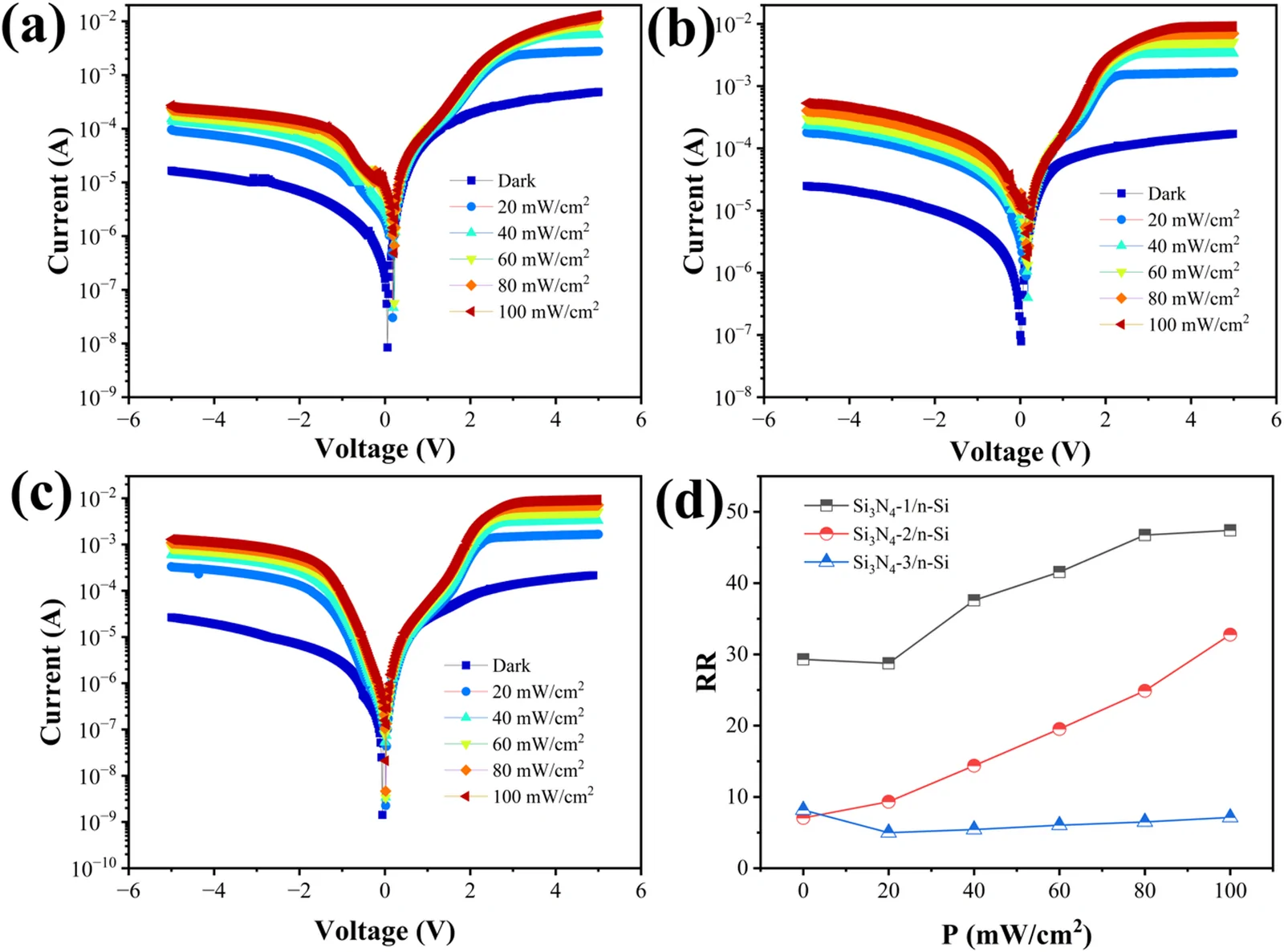
Semi-logarithmic current-voltage (I–V) characteristics of (**a**) Si_3_N_4_-1/n-Si, (**b**) Si_3_N_4_-2/n-Si, and (**c**) Si_3_N_4_-3/n-Si hybrid photodiodes evaluated under dark and different illumination intensities (20–100 mW/cm^2^) at room temperature. (**d**) RR variation vs. light intensity (P) for three hybrid photodiodes at ± 5 V.

The electrical performance of the manufactured Si_3_N_4_ hybrid photodiodes was examined utilizing current-voltage (I-V) measurements under dark conditions and various light intensities at room temperature. The measured I–V curves are presented in Fig. 3a–c for Si_3_N_4_-1/n-Si, Si_3_N_4_-2/n-Si, and Si_3_N_4_-3/n-Si devices, respectively. All photodiodes exhibited clear rectifying behavior, with forward current increasing exponentially as the applied voltage increased. Upon illumination, a significant enhancement in reverse-bias photocurrent was observed compared to the dark condition, and the photocurrent increased proportionally with light intensity. In contrast, the forward-bias current showed minimal dependence on illumination. This pronounced increase in reverse photocurrent confirms the photosensitive nature of the Si_3_N_4_-based hybrid photodiodes, indicating their suitability for optoelectronic applications such as photodetectors and photodiodes.

The observed photoresponse originates from the generation of electron–hole pairs within the hybrid structure under illumination. The interaction between semiconductor materials and incident photons depends on the electronic excitation processes within the material^22^. Understanding the occupation and transport of these excited states is essential for optimizing hybrid optoelectronic device performance. In general, semiconductor materials possess energy levels analogous to the highest occupied molecular orbital (HOMO) and the lowest unoccupied molecular orbital (LUMO), which define the bandgap and govern charge transport behavior. The distribution of these energy states is influenced by factors such as molecular interaction, structural disorder, and material composition^23^. Under applied bias or optical excitation, electrons in lower energy states can be promoted to higher energy levels, facilitating charge transport through the device^24^. According to quantum mechanical principles, including the Pauli exclusion principle, electron excitation and occupancy in these energy states follow specific allowed configurations, which determine the electrical and optical properties of the hybrid system^25^ as follows:

1 $$\left\{ {\begin{array}{*{20}{c}}{\left| {11} \right\rangle = \uparrow \: \uparrow \:\:\left( {\rm{a}} \right)}\\{\:\left| {10} \right\rangle = \frac{1}{{\sqrt 2 }}( \uparrow \: \downarrow \: + \: \downarrow \: \uparrow \:)\:\left( {\rm{b}} \right)}\\{\left| {1\: - \:1} \right\rangle = \downarrow \: \downarrow \:\:\left( c \right)}\\{\:\left| {00} \right\rangle = \frac{1}{{\sqrt 2 }}\left( { \uparrow \: \downarrow \:\: - \: \downarrow \: \uparrow \:} \right)\left( d \right)}\end{array}} \right\}$$

Among these configurations, the first three states, labeled (a), (b), and (c), correspond to the triplet state (s = 1), whereas configuration (d) represents the singlet state (s = 0). Under illumination, the Si_3_N_4_ layer absorbs incident photons, resulting in the excitation of an electron to the singlet excited state (S_1_) and leaving a hole in the ground singlet state (S_o_). Alternatively, if the incident light is not absorbed by the Si_3_N_4_ layer, it can penetrate and excite charge carriers in the Si substrate. This process enables efficient separation of photogenerated electrons and holes at the Si_3_N_4_/Si interface, reducing recombination probability and enhancing charge separation efficiency.

As shown in Fig. 3a–c, the reverse-bias photocurrent increases significantly under illumination and continues to rise with increasing light intensity, demonstrating typical photodiode characteristics. The electrical performance of the manufactured Si_3_N_4_ hybrid photodiodes was examined utilizing current–voltage (I–V) measurements under dark conditions and various light intensities at room temperature.

At a reverse bias of 5 V, the dark current of the Si_3_N_4_-1/n-Si photodiode was measured as 2.8 × 10^−5^ A, which increased to approximately 1.10 × 10^−4^ A and 5.30 × 10^−4^ A under illumination intensities of 20 and 100 mW/cm^2^, respectively. Similarly, the Si_3_N_4_-2/n-Si device exhibited a dark current of 3.81 × 10^−5^ A, which increased to 3.78 × 10^−4^ A and 7.96 × 10^−4^ A under the same illumination conditions. For the Si_3_N_4_-3/n-Si photodiode, the dark current was 4.69 × 10^−5^ A, and the corresponding photocurrents increased to 6.30 × 10^−4^ A and 1.15 × 10^−3^ A at 20 and 100 mW/cm^2^, respectively. In all cases, the reverse-bias photocurrent increased with illumination and eventually approached saturation at higher light intensities.

The current–voltage (I–V) method is widely used to assess the electrical properties of semiconductor devices and comprehend their charge transport mechanisms. Figure 3a–c shows the semi-logarithmic I–V characteristics of hybrid-based n-Si photodiodes recorded in dark and under illumination with light intensity increments of 20 mW cm^−2^ at room temperature (300 K). The gadget shows evident rectifying activity in the forward bias area (V ≤ 1.0 V), indicating the creation of a junction. Higher voltages (V ≥ 1.0 V) cause divergence from linearity due to series resistance (R_s_). Electrical characteristics, including ideality factor (n), barrier height ($\:{\varPhi\:}_{B}$), R_s_, and reverse saturation current (I_o_), were calculated from I–V data using TE, Cheung, and Norde methods. The dark I–V features of the fabricated devices can be characterized using the thermionic emission (TE) theory^26^. The linear area of semi-logarithmic forward bias, ln(I)–V plots, was utilized to derive essential diode parameters, such as $\:{\varPhi\:}_{B}$, n, and I_o_ using TE theory. According to this model, charge transport occurs via thermally activated carriers overcoming the potential barrier at the junction, and the current–voltage relationship can be expressed as follows^27^:

2 $$\:I={I}_{0}[\mathrm{e}\mathrm{x}\mathrm{p}(\frac{\mathrm{q}\mathrm{V}}{\mathrm{n}\mathrm{k}\mathrm{T}\:})-1]$$

where I_0_ is given as

3 $$\:{I}_{0}={AA}^{*}{T}^{2}\mathrm{e}\mathrm{x}\mathrm{p}(-\frac{\mathrm{q}{\varPhi\:}_{B}}{\mathrm{k}\mathrm{T}\:})$$

where q is the electronic charge (1.6 × 10^− 19^ C), and k is the Boltzmann constant (1.381 × 10^− 23^ J/K). A, A*, and $\:{\varPhi\:}_{B}$ the contact area (7.85 × 10^− 3^ cm^2^), Richardson constant (112 A cm^− 2^ K^2^ for *n*-type Si), and barrier height, respectively. The parameters *n* and *Φ*_bo_ can be calculated using Eqs. (3) and (4)^28^:

4 $$\:n\:=\frac{q}{\mathrm{k}\mathrm{T}\:}\mathrm{e}\mathrm{x}\mathrm{p}\left(\frac{\mathrm{d}\mathrm{V}}{\mathrm{d}\mathrm{l}\mathrm{n}\mathrm{I}}\right)$$

5 $$\:{\varPhi\:}_{B}=\frac{\mathrm{k}\mathrm{T}}{\mathrm{q}\:}\mathrm{l}\mathrm{n}\left(\frac{{AA}^{*}{T}^{2}}{{I}_{0}}\right)$$

The ideality factor (n) is an important parameter that reflects the quality and transport mechanism of the junction. For an ideal diode, n equals unity, whereas values greater than one indicate non-ideal behavior caused by factors such as interface defects, surface irregularities, barrier inhomogeneity, image force lowering, tunneling effects, and high series resistance^29^. These deviations are often related to fabrication conditions and material quality^30^.

The rectification ratio (RR), defined as the ratio of forward current to reverse current at the same voltage (RR = IF/IR)^31^, is a key indicator of diode performance and junction quality. The variation of RR with light intensity for the Si_3_N_4_-1/n-Si, Si_3_N_4_-2/n-Si, and Si_3_N_4_-3/n-Si photodiodes at ± 5 V is presented in Fig. 3d. Under dark conditions, the RR values were approximately 29, 7, and 8 for Si_3_N_4_-1/n-Si, Si_3_N_4_-2/n-Si, and Si_3_N_4_-3/n-Si, respectively. With increasing illumination intensity, the RR increased significantly, reaching values as high as 46 for the Si_3_N_4_-1/n-Si device.

This enhancement is attributed to the increased generation of electron–hole pairs under illumination, which raises the reverse photocurrent due to the drift of photogenerated carriers. As a result, the difference between forward and reverse currents becomes more pronounced, improving the rectifying behavior. The observed increase in RR with light intensity confirms the strong photosensitivity and efficient charge separation of the Si_3_N_4_-based hybrid photodiodes. Minor fluctuations observed at certain illumination levels are likely due to measurement noise or experimental uncertainties^28^. Overall, the high RR values demonstrate that the fabricated devices exhibit good rectification characteristics and are suitable for photo detector applications.

**Fig. 4:**
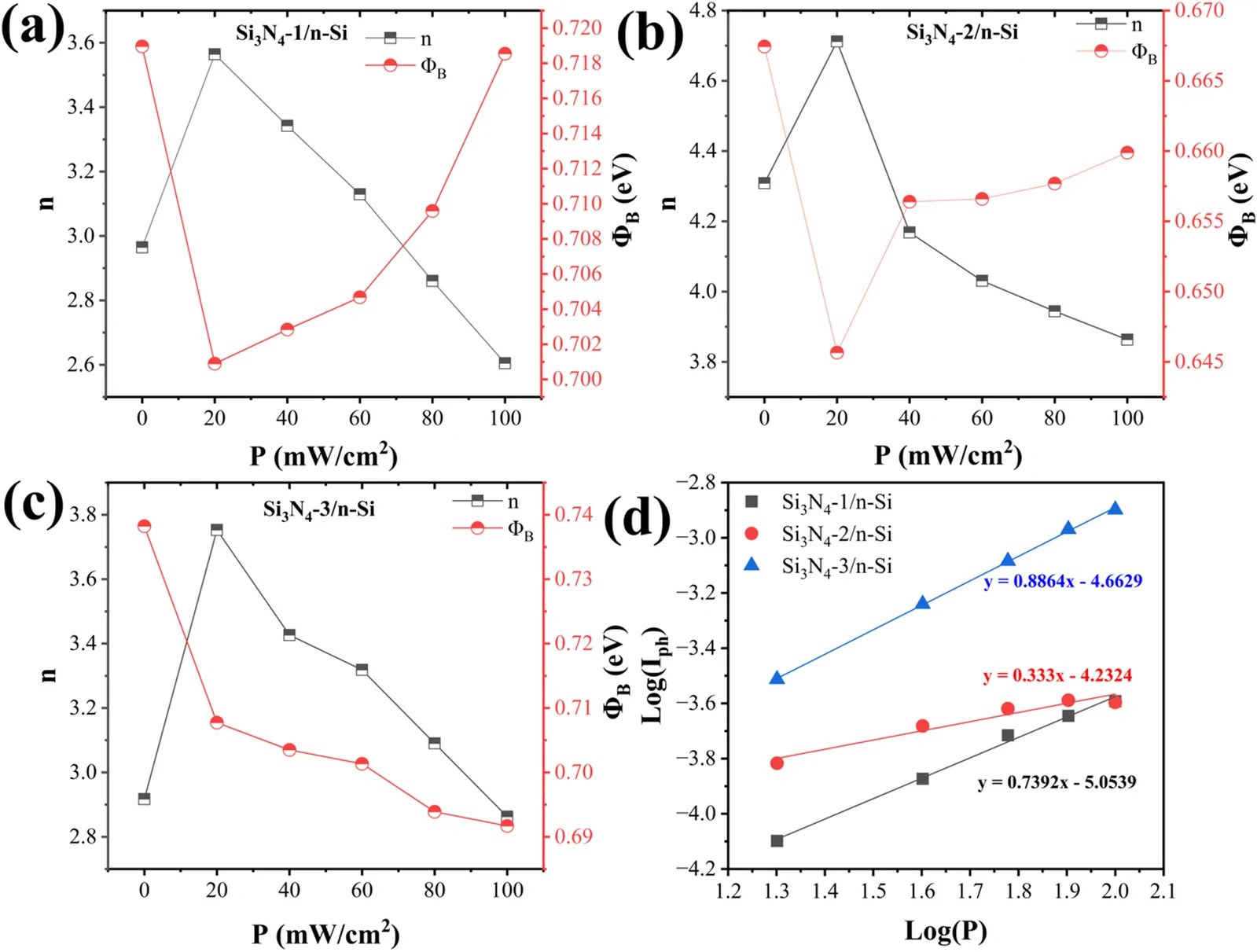
Variation of ideality factor (n) and barrier height ($\:{\varPhi\:}_{B}$) as a function of light intensity (P) for (**a**) Si_3_N_4_-1/n-Si, (**b**) Si_3_N_4_-2/n-Si, and (**c**) Si_3_N_4_-3/n-Si hybrid photodiodes. (**d**) Log–log plot of photocurrent ($\:{I}_{ph}$) versus light intensity (P) for the three devices, including linear fitting curves.

The n and $\:{\varPhi\:}_{B}$ values were extracted from the slope and intercept of the linear regions of the forward bias ln(I)–V characteristics using Eqs. (3) and (4) for the Si_3_N_4_-based hybrid photodiodes (Fig. 4a–c). The variation of n and $\:{\varPhi\:}_{B}\:$as a function of light intensity is presented in Fig. 4a–c. As shown in Fig. 4a,b, n decreases with increasing illumination intensity, while $\:{\varPhi\:}_{B}$ increases. This behavior is attributed to enhanced photocarrier generation and improved carrier transport at the metal–semiconductor interface, thereby reducing recombination effects and improving junction uniformity. Such illumination-dependent improvement in barrier height and reduction in ideality factor have been widely reported for Schottky and hybrid photodiodes^32–35^.

According to thermionic emission theory, an ideal diode has *n* = 1. However, the obtained *n* > 1 values indicate the presence of interface states, series resistance, and barrier inhomogeneities. The incorporation of the Si_3_N_4_ interfacial layer modifies the interface potential profile and reduces inhomogeneous barrier distribution, thereby improving device performance. In contrast, Fig. 4c shows that both n and $\:{\varPhi\:}_{B}$ decrease with illumination intensity for the Si_3_N_4_-3/n-Si device, which may be attributed to enhanced interface state participation and trap-assisted transport under illumination.

The photoconduction mechanism was analyzed using the photocurrent–illumination intensity relationship given by Eq. (5):

6 $$\:{I}_{ph}={CP}^{m}$$

where $\:{I}_{ph}$ is photocurrent, * C* is a constant number, * m* is an exponent value, which gives information about the photoconduction mechanism, and P is the illumination intensity. The log($\:{I}_{ph}$)–log(P) plots shown in Fig. 4d exhibit excellent linearity, confirming stable photoconduction behavior. The m values obtained from the slopes are 0.7392, 0.333, and 0.8864 for Si_3_N_4_-1/n-Si, Si_3_N_4_-2/n-Si, and Si_3_N_4_-3/n-Si devices, respectively. The m values between 0.5 and 1 indicate that the photocurrent is governed by photogeneration with the presence of localized trap states in the mobility gap^34,36,37^. The higher m value observed for the Si_3_N_4_-3/n-Si device suggests reduced trap density and more efficient carrier transport, demonstrating the critical role of the Si_3_N_4_ interfacial layer in controlling the photodiode performance.

The electrical transport parameters of the Si_3_N_4_-1/n-Si, Si_3_N_4_-2/n-Si, and Si_3_N_4_-3/n-Si photodiodes were further evaluated using the Cheung and Cheung method, which is widely employed to extract R_s_, n, and $\:{\varPhi\:}_{B}$ from forward bias I–V characteristics^38–41^. According to this method, the dV/dln(I) versus I and H(I) versus I plots exhibit linear behavior when thermionic emission (TE) is the dominant conduction mechanism. This method provides a reliable estimation of diode parameters and allows evaluation of interface quality and carrier transport mechanisms^38,39^.

**Fig. 5:**
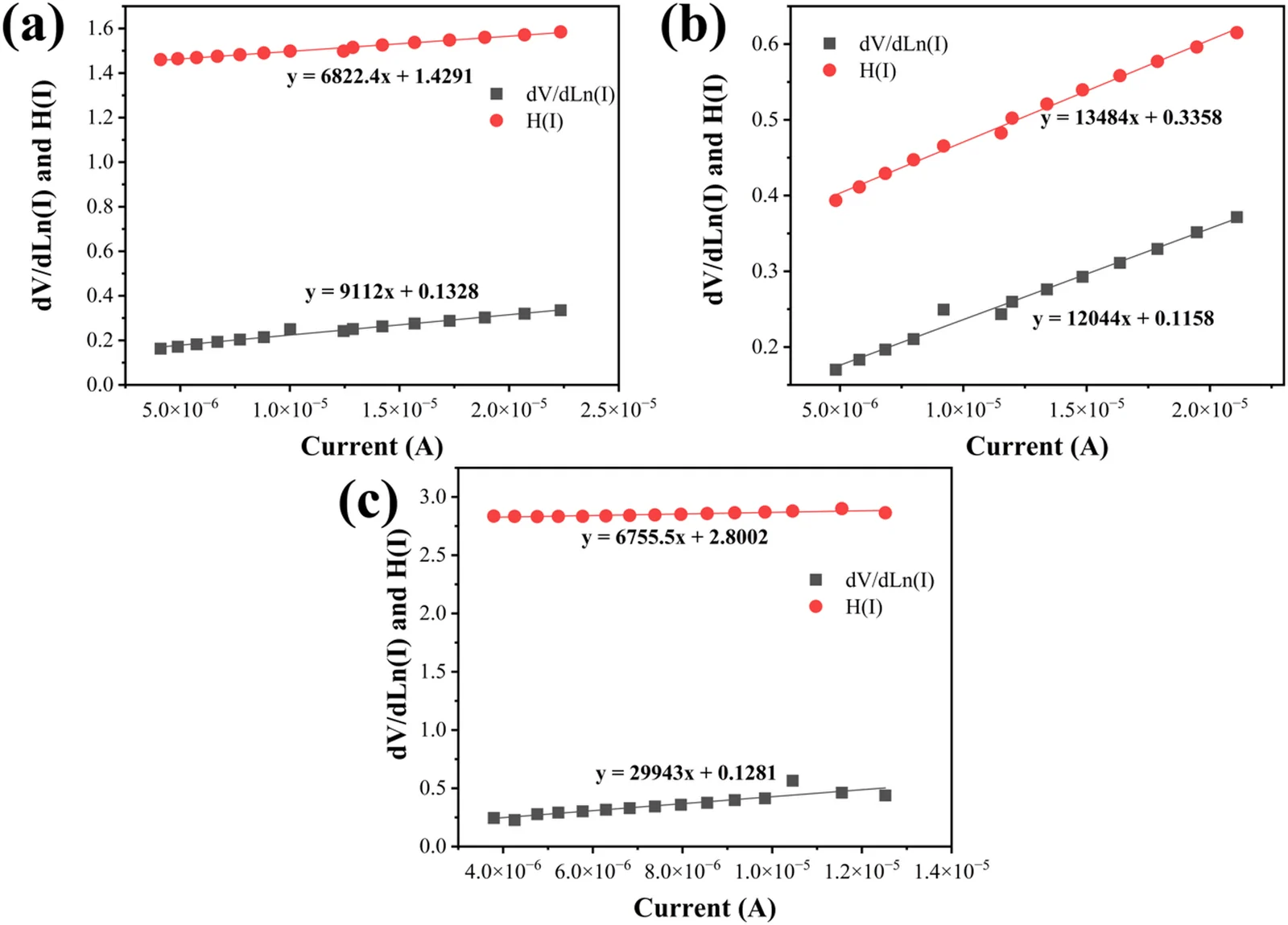
Cheung and Cheung’s plots of dV/dln(I) versus I and H(I) versus I for (**a**) Si_3_N_4_-1/n-Si, (**b**) Si_3_N_4_-2/n-Si, and (**c**) Si_3_N_4_-3/n-Si photodiodes under forward bias. The linear trends in both plots support the applicability of thermionic emission theory. From the dV/dln(I)–I plot, the slope corresponds to the series resistance (R_s_) and the intercept gives the ideality factor (n). In the H(I)–I plot, the slope also represents R_s_, while the intercept is used to estimate the barrier height (Φ_B_). These results indicate that the Si_3_N_4_ interfacial layer significantly affects charge transport and interface properties.

As shown in Fig. 5a–c, both dV/dln(I) and H(I) vary linearly with current for all devices, confirming that thermionic emission is the dominant transport mechanism in the intermediate forward bias region. The slope of the dV/dln(I)–I plots provides the series resistance, while the intercept corresponds to nkT/q, allowing the determination of the n. Similarly, the slope of the H(I)–I plots also gives the R_s_, and the intercept provides the $\:{\varPhi\:}_{B}$.

For the Si_3_N_4_-1/n-Si device (Fig. 5a), the linear characteristics indicate relatively stable carrier transport across the interface. However, the slope values suggest a moderate series resistance, which may be attributed to interface states and interfacial layer thickness. The presence of the Si_3_N_4_ layer improves junction uniformity while still maintaining some interface inhomogeneity.

In the case of the Si_3_N_4_-2/n-Si device (Fig. 5b), the improved linearity and modified slope indicate improved interface quality and reduced recombination losses. The reduction in series resistance compared to Si_3_N_4_-1/n-Si suggests enhanced carrier transport efficiency due to better interface passivation provided by the Si_3_N_4_ interlayer.

For the Si_3_N_4_-3/n-Si device (Fig. 5c), the linear behavior remains valid, confirming thermionic emission conduction. However, the higher slope observed in the dV/dln(I) plot indicates an increase in series resistance. This increase may be attributed to the thicker or more resistive interfacial layer, which introduces additional resistance to carrier transport. Nevertheless, the strong linearity of both plots confirms that the Cheung method is applicable and that the Si_3_N_4_ interfacial layer significantly influences the electrical parameters of the device.

Overall, the Cheung analysis confirms that the interfacial Si_3_N_4_ layer plays a critical role in controlling the series resistance and interface properties of the photodiodes. Proper optimization of the interfacial layer improves junction quality, reduces interface state density, and enhances carrier transport across the metal–semiconductor interface.

**Fig. 6:**
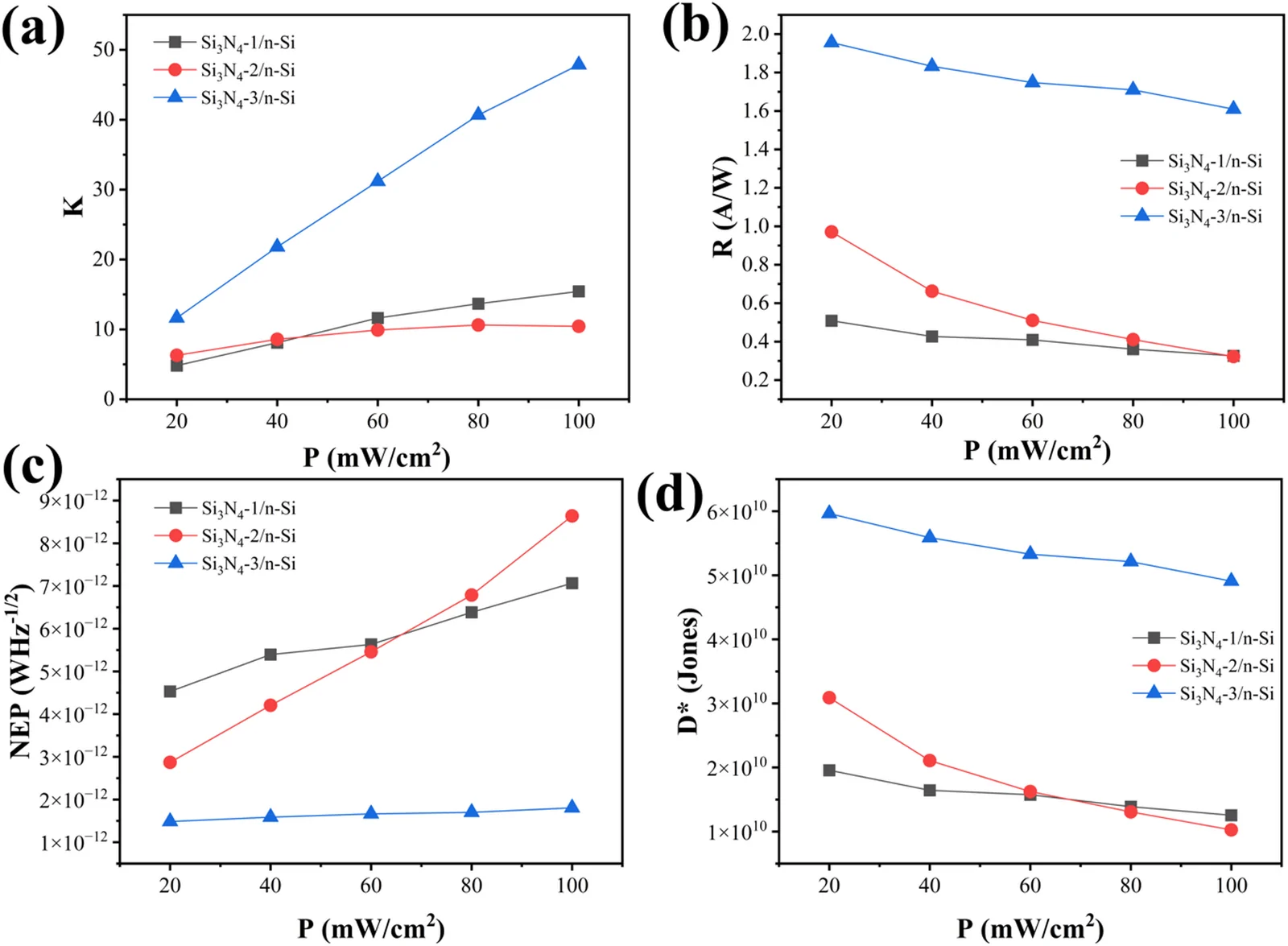
Variation of the photodetector performance parameters with illumination power density (P) for the Si_3_N_4_-1/n-Si, Si_3_N_4_-2/n-Si, and Si_3_N_4_-3/n-Si hybrid photodiodes: (**a**) photosensitivity (K) versus illumination intensity, (**b**) responsivity (R) versus illumination intensity, (**c**) noise equivalent power (NEP) versus illumination intensity, and (**d**) specific detectivity (D*) versus illumination intensity. The plots demonstrate the influence of the Si_3_N_4_ interfacial layer thickness on the photoresponse and sensitivity characteristics of the fabricated photodiodes.

The illumination-dependent optoelectronic performance parameters of the Si_3_N_4_-1/n-Si, Si_3_N_4_-2/n-Si, and Si_3_N_4_-3/n-Si hybrid photodiodes are presented in Fig. 6a–d. These parameters provide important insight into the sensitivity, efficiency, and detection capability of the fabricated devices. Figure 6a shows the variation of photosensitivity (K) as a function of illumination intensity. K is calculated using the following equation^42^:

7 $$\:\mathrm{K}=\frac{{I}_{ph}}{{I}_{dark}}\:\:$$

For all devices, K increases with increasing light power density. This behavior occurs because higher illumination generates a larger number of photogenerated electron–hole pairs, resulting in a higher photocurrent relative to the dark current. Among the devices, the Si_3_N_4_-3/n-Si photodiode exhibits the highest photosensitivity, indicating more efficient carrier generation and separation at the interface. The improvement in photosensitivity can be attributed to the enhanced passivation and reduced recombination at the metal–semiconductor interface due to the presence of the Si_3_N_4_ interfacial layer. Similar increases in photosensitivity with increasing illumination intensity have been widely reported for hybrid photodiodes and photoconductive devices^43^. The variation of responsivity with illumination intensity is shown in Fig. 6b. Responsivity decreases gradually as the illumination intensity increases for all devices. Responsivity represents the ratio of generated photocurrent to the incident optical power and reflects the photoelectric conversion efficiency of the photodetector^44^:

8 $$\:R=\frac{{I}_{ph}-{I}_{dark}}{\mathrm{P}\mathrm{A}}\:\:$$

where A represents the diode active detector area.

The observed decrease in responsivity at higher illumination intensities is commonly attributed to photocarrier recombination, trap filling, and saturation effects, which limit the extraction efficiency of photogenerated carriers. Under strong illumination, the recombination rate increases and the carrier lifetime may decrease, resulting in reduced responsivity. Such behavior has been reported in many semiconductor photodetectors and is generally associated with trap-assisted recombination and carrier transport saturation^17^. Among the studied devices, the Si_3_N_4_-3/n-Si structure exhibits the highest responsivity, suggesting improved carrier collection efficiency. Figure 6c presents the variation of noise equivalent power (NEP) with illumination intensity. The NEP values increase with increasing light intensity for all devices. NEP represents the minimum detectable optical power that produces a signal equal to the noise level of the detector. Therefore, a lower NEP indicates higher detector sensitivity^45^. The relatively low NEP values observed for the Si_3_N_4_-3/n-Si device indicate improved signal-to-noise performance compared with the other structures. The increase in NEP with increasing illumination may be related to enhanced noise currents resulting from increased carrier recombination and transport fluctuations under stronger illumination. The device’s performance under light can be better understood by calculating its specific detectivity (D*), and photocurrent (I_ph_) using transient photocurrent studies. Equation^46^ can be used to calculate these characteristics using n- and p-type Si. Figure 6d shows the variation of specific detectivity (D*) with illumination intensity. Detectivity is a key figure of merit for photodetectors that quantifies their ability to detect weak optical signals and is inversely proportional to NEP^47^. The NEP and parameters are calculated using Eqs. (8) and (9) respectively:

9 $$\:NEP=\:\frac{\sqrt{2\mathrm{q}{I}_{d}}}{R}$$

10 $$\:{D}^{*}=\:R\sqrt{\frac{A}{2\mathrm{q}{I}_{d}}}\:\:$$

The analytical expressions used to evaluate the optoelectronic parameters of the Si_3_N_4_/n-Si devices are consistent with established models in semiconductor device physics. Similar formulations for current density, responsivity, and detectivity have been reported in recent literature, confirming the validity of the adopted approach^48,49^. As shown in the figure, D* decreases gradually with increasing illumination intensity for all devices, which is consistent with the increase in NEP and the decrease in responsivity observed at higher light intensities. The Si_3_N_4_-3/n-Si device exhibits the highest detectivity, reaching values on the order of 10^10^ Jones, indicating superior sensitivity and improved photodetection capability compared with the other devices.

Overall, the results demonstrate that the insertion of the Si_3_N_4_ interfacial layer significantly influences the optoelectronic performance of the photodiodes. Specifically, the Si_3_N_4_-3/n-Si device performs the best overall, with comparatively low NEP values and the maximum photosensitivity, responsivity, and detectivity. These enhancements imply that the improved Si_3_N_4_ interfacial layer increases carrier transport, reduces recombination losses, and boosts the hybrid photodiode structure’s photoresponse.

**Fig. 7:**
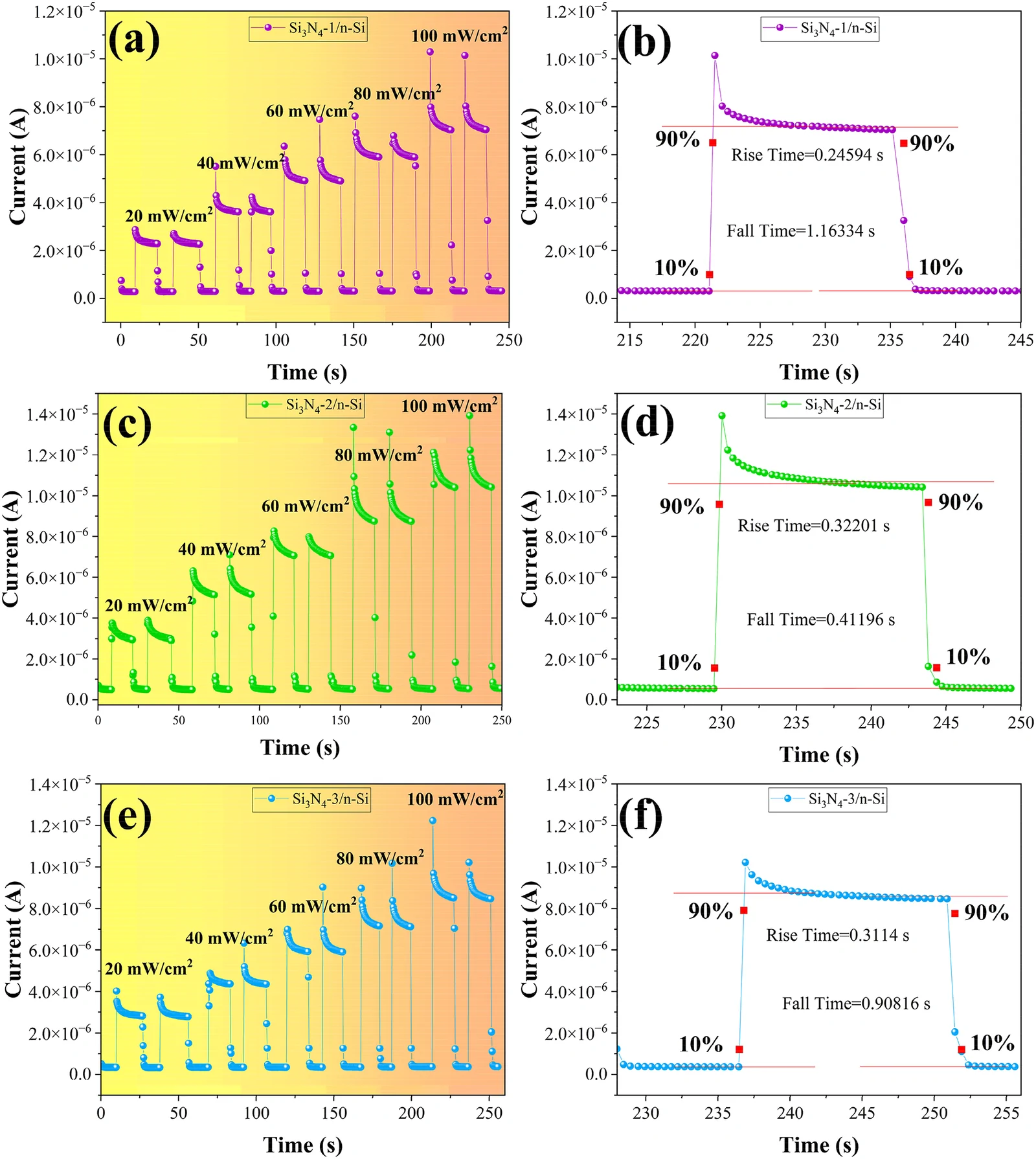
Time-dependent photoresponse of fabricated Si_3_N_4_/n-Si photodiodes under periodic illumination (20–100 mW/cm^2^): (**a**,**c**,**e**) I–t characteristics of Si_3_N_4_-1/n-Si, Si_3_N_4_-2/n-Si, and Si_3_N_4_-3/n-Si devices, respectively, and (**b**,**d**,**f**) their corresponding rise and fall time measurements at 100 mW/cm^2^.

To investigate the relationship between photocurrent and light intensity, the photoresponse of the manufactured hybrid photodiodes is investigated at various light intensities (Fig. 7). The dynamic photoresponse behavior of the Si_3_N_4_-1/n-Si, Si_3_N_4_-2/n-Si, and Si_3_N_4_-3/n-Si hybrid photodiodes was investigated using time-dependent current (I–t) measurements under periodic illumination at different power densities ranging from 20 to 100 mW/cm^2^, as shown in Fig. 7a,c,e. Similar stable ON/OFF switching behavior has been widely reported for Si-based and hybrid photodiodes^17^.

The photocurrent rises systematically with illumination intensity, owing to increased photogeneration of electron-hole pairs and greater carrier transport at higher light intensities. This pattern is similar to previously reported Si-based Schottky and heterojunction photodetectors, in which photocurrent increases with light intensity due to increased carrier production^50^.

Among the three structures, the Si_3_N_4_-3/n-Si photodiode exhibits the highest photocurrent response, followed by the Si_3_N_4_-2/n-Si and Si_3_N_4_-1/n-Si devices. The improved photoresponse can be attributed to the presence of the Si_3_N_4_ interfacial layer, which enhances the separation and transport of photogenerated carriers while suppressing recombination at the metal-semiconductor interface. The interfacial layer also helps reduce interface trap states and improve junction quality, thereby facilitating efficient carrier transport across the junction. Interface modification has been shown to significantly improve carrier separation efficiency and overall photodetector performance^51^.

Furthermore, repeated ON/OFF switching cycles yield approximately the same photocurrent levels across illumination phases, demonstrating that the photodiodes are stable and reproducible. The rapid increase and subsequent decline of the photocurrent indicate that photogenerated carriers are efficiently generated and recombined within the device structure. Such characteristics are essential for practical photodetector applications and are consistent with recent reports on high-performance hybrid and Si-based photodetectors^52^.

The rise time and fall time values of the fabricated devices were determined at an illumination intensity of 100 mW/cm^2^ and are presented in Fig. 7b,d,f for the Si_3_N_4_-1/n-Si, Si_3_N_4_-2/n-Si, and Si_3_N_4_-3/n-Si devices, respectively. The rise time is defined as the time required for the photocurrent to increase from 10% to 90% of its maximum value under light illumination, whereas the fall time corresponds to the time taken for the photocurrent to decay from 90% to 10% after the light source is switched off. The extracted rise/fall times for the Si_3_N_4_-1/n-Si device were found to be 245.94 ms and 1163.34 ms, respectively. Similarly, the Si_3_N_4_-2/n-Si device exhibited rise and fall times of 322.01 ms and 411.96 ms, while the Si_3_N_4_-3/n-Si device showed rise and fall times of 311.4 ms and 908.16 ms, respectively. Among the fabricated devices, the Si_3_N_4_-2/n-Si photodetector demonstrated the fastest decay response with the lowest fall time, indicating more efficient carrier recombination and extraction dynamics. In contrast, the Si_3_N_4_-1/n-Si device exhibited the quickest photo-generation response due to its comparatively lower rise time. Overall, the obtained response times are in the millisecond range, confirming the good switching behavior and stable photoresponse characteristics of the fabricated Si_3_N_4_/n-Si heterostructure photodetectors.

The Si_3_N_4_ interfacial layer improves the photoresponse performance of hybrid photodiodes. The Si_3_N_4_-3/n-Si device exhibits the strongest and most stable photocurrent response, indicating that the optimized interfacial layer enhances carrier separation and transport, thereby improving photodetection performance.

**Fig. 8:**
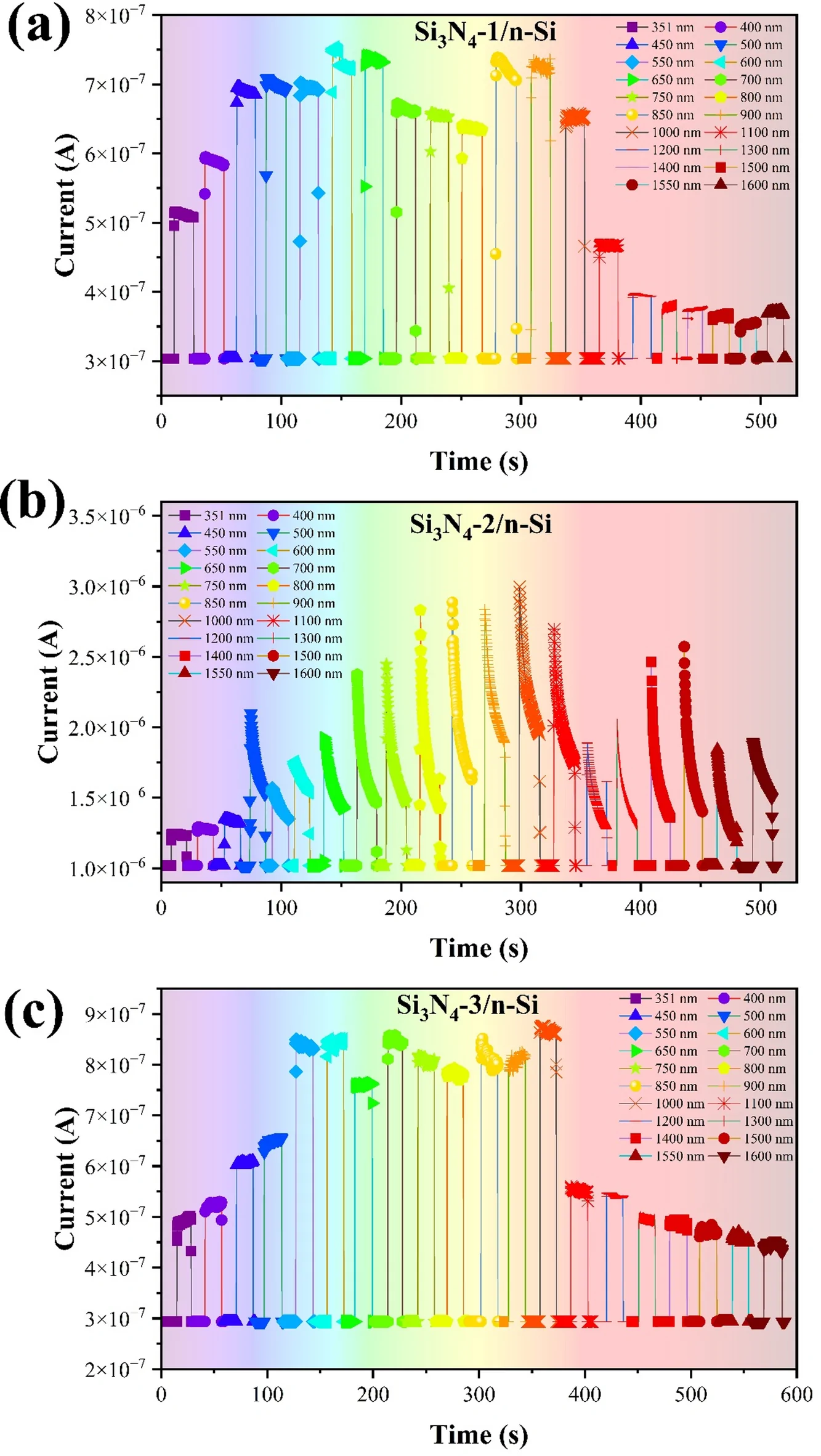
Wavelength-dependent time-resolved photoresponse (I–t) characteristics of the hybrid photodiodes under monochromatic illumination in the 351–1600 nm spectral range: (**a**) Si_3_N_4_-1/n-Si, (**b**) Si_3_N_4_-2/n-Si, and (**c**) Si_3_N_4_-3/n-Si devices. The plots illustrate the spectral sensitivity and switching behavior of the devices across the UV–visible–near infrared (UV–Vis–NIR) region, highlighting the influence of the Si_3_N_4_ interfacial layer on wavelength-selective photoresponse.

The photoresponse of the Si_3_N_4_-1/n-Si, Si_3_N_4_-2/n-Si, and Si_3_N_4_-3/n-Si hybrid photodiodes was examined using time-resolved current (I-t) measurements under monochromatic irradiation from 351 to 1600 nm, as shown in Fig. 8a–c. The devices display clear and repeated ON/OFF switching behavior at each wavelength, proving stable and reliable photodetection over a wide spectral range.

For all devices, the photocurrent is strongly dependent on the incident wavelength. The photoresponse increases dramatically in the visible range (≈ 400–800 nm) and gradually decreases in the near-infrared (NIR) area (> 1100 nm). Silicon’s intrinsic bandgap (~ 1.12 eV) limits effective photon absorption at wavelengths below ~ 1100 nm. Beyond this range, silicon’s absorption coefficient reduces dramatically, resulting in lower photocurrent generation. Similar wavelength-dependent behavior has been commonly seen in Si-based photodetectors^53,54^.

Despite this constraint, detectable photocurrent is still seen in the NIR region (1100–1600 nm). This can be attributed to sub-bandgap absorption mechanisms, interface states, and defect-assisted carrier production introduced by the interfacial Si_3_N_4_ layer. Such extended spectrum response has been observed in tailored silicon photodetectors and hybrid devices, where interface alteration or defect states allow NIR detection beyond the usual bandgap limit^53,55^.

Among the studied devices, the Si_3_N_4_-3/n-Si photodiode has the maximum photocurrent across all spectral ranges, followed by Si_3_N_4_-2/n-Si and Si_3_N_4_-1/n-Si structures. The improved Si_3_N_4_ interfacial layer improves light absorption and carrier collection efficiency, minimizing recombination losses. The interfacial layer also plays an important function in changing energy band alignment and enhancing charge separation, resulting in increased spectral response. Similar improvements due to interface engineering and nanostructuring have been reported in recent silicon-based photo detectors^54,56^.

Furthermore, the constant ON/OFF switching behavior seen at all wavelengths indicates that the devices are stable and reproducible. The photocurrent’s comparatively rapid increase and decay imply effective photocarrier generation and recombination dynamics, which are required for broadband photodetection applications. Figure 8b shows a sharp and immediate increase in photocurrent upon illumination, indicating a rapid photoresponse behavior. The photocurrent increases progressively with increasing light intensity, confirming the device sensitivity to incident light power density. Similar behavior has also been reported in previous studies^57^. This I–t characteristics also exhibit capacitive behavior, evidenced by the sharp photocurrent peaks followed by gradual stabilization under continuous illumination. This behavior is associated with charge accumulation, trapping/de-trapping processes, and interfacial carrier storage within the device. Similar capacitive photoresponse characteristics have also been reported in previous studies on halide perovskite photodetectors^58^.

The results show that adding the Si_3_N_4_ interfacial layer considerably improves the devices’ broadband spectrum response. The Si_3_N_4_-3/n-Si photodiode is a viable contender for broadband photodetector applications due to its exceptional UV–Vis-NIR performance.

**Fig. 9:**
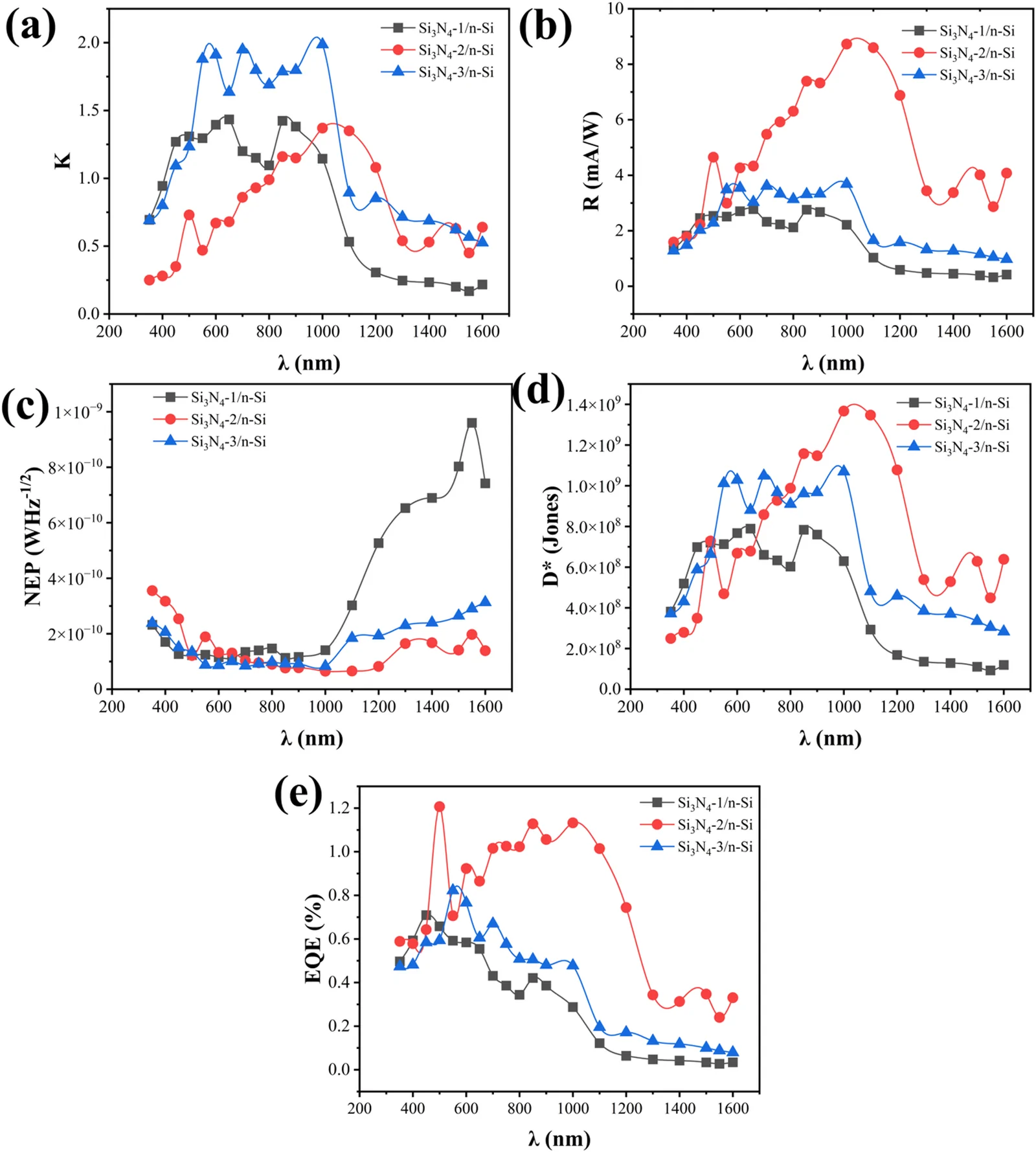
Wavelength-dependent optoelectronic performance parameters of the Si_3_N_4_-based hybrid photodiodes: (**a**) photosensitivity (K), (**b**) responsivity (R), (**c**) noise equivalent power (NEP), (**d**) specific detectivity (D*), and (**e**) external quantum efficiency (EQE) for Si_3_N_4_-1/n-Si, Si_3_N_4_-2/n-Si, and Si_3_N_4_-3/n-Si devices. The graphs demonstrate the spectrum performance and impact of the Si_3_N_4_ interfacial layer on broadband photodetection properties.

Parameters including $\:{I}_{ph}$, K, R, NEP, and D* are the primary variables that affect a photo detector’s effectiveness. The quantity of charge carriers gathered by the corresponding electrodes for each incident photon is typically estimated by EQE. It can be calculated using the following relation based on responsivity^59^:

11 $$\:EQE=\frac{Rhc}{q\lambda\:}$$

where h is Planck’s constant (6.6 × 10^− 34^ J/s), c is the velocity of light (3.0 × 10^8^ m/s), λ is the wavelength of incident radiation, and Rλ is the photoresponsivity at specific light wavelengths. The ratio of excited electrons to photons that make it to the detector is measured by the EQE. The wavelength-dependent optoelectronic performance parameters of the fabricated photodiodes are presented in Fig. 9a–e. These results provide a comprehensive understanding of the spectral sensitivity and detection capability of the Si_3_N_4_-based hybrid structures.

Figure 9a shows that K is strongly dependent on wavelength, with peak values in the visible area (about 500–900 nm). This pattern is consistent with silicon’s high absorption coefficient in the visible spectrum. The Si_3_N_4_-3/n-Si device has the greatest K values, indicating superior photocarrier production and interface quality. The drop in K at longer wavelengths (> 1100 nm) is ascribed to lower photon absorption caused by the silicon bandgap constraint. Similar spectrum behavior has been seen in silicon-based photodetectors^60,61^.

Figure 9b demonstrates that responsivity follows a similar trend, increasing from UV to visible range and reaching a maximum near ~ 900–1000 nm before declining towards the NIR region. This pattern illustrates Silicon’s wavelength-dependent absorption and carrier production efficiency. The Si_3_N_4_-2/n-Si device has the maximum responsivity, indicating optimal carrier collection and minimized recombination. The decrease in responsivity beyond ~ 1100 nm is consistent with decreasing absorption in Si-based devices^60,62^.

The variation of NEP with wavelength is presented in Fig. 9c. The NEP values are lowest in the visible region, indicating high sensitivity, and increase significantly in the NIR region due to reduced responsivity and increased noise contributions. Since NEP is inversely proportional to detector sensitivity, the lower NEP values observed for the Si_3_N_4_-2/n-Si device confirm its superior detection capability in the visible range. This trend is consistent with recent studies on broadband photodetectors^10,62^.

Figure 9d depicts the fluctuation in particular D* with wavelength. The D* values perform best in the visible-near infrared transition area (~ 800–1100 nm), when responsivity and signal-to-noise ratio are improved. The Si_3_N_4_-2/n-Si device has the highest detectivity (~ 10^5^ Jones), indicating superior sensitivity. The drop in D* at longer wavelengths is attributable to lower photocurrent production and higher noise levels. Similar behavior has been observed in high-performance Si-based and hybrid photodetectors^10,61,62^.

The EQE spectra in Fig. 9e exhibit similar trends to responsivity, with peak values in the visible range and a gradual drop towards the NIR region. The Si_3_N_4_-2/n-Si device has the highest EQE, which indicates efficient photon-to-electron conversion. The decline in EQE at longer wavelengths is consistent with silicon’s low absorption capabilities beyond its bandgap. The link between EQE and responsivity supports the experimental findings.

Table 1 compares the photoresponse performance of different interlayer-based photodetectors reported in the literature with the Si_3_N_4_-based device developed in this work. The comparison includes key parameters such as operating wavelength, R, D*, EQE, response time, and applied bias voltage. Previously reported devices, including Ta_2_O_5_/MWCNT, GeSe/WS_2_/MoS_2_, SiO_2_/Si_3_N_4_, Si_3_N_4_/Al_2_O_3_/ReS_2_, and rGO/Si_3_N_4_/GaAs, demonstrated notable responsivity and detectivity values under different operating conditions. Among these devices, p-GaN/n-ZnO, GeSe/WS_2_/MoS_2_, SnSe_2_/PEDOT: PSS, rGO/Si_3_N_4_/GaAs, and the Si_3_N_4_-2 device developed in this work operated under self-powered (0 V bias) conditions, which is highly desirable for low-power and energy-efficient optoelectronic applications.

In particular, the Si_3_N_4_/Al_2_O_3_/ReS_2_ device exhibited the highest responsivity of 2470 mA/W under an applied bias of 10 V, while the SiO_2_/Si_3_N_4_ heterostructure achieved the highest detectivity of 2.26 × 10^10^ Jones and an EQE of 70.68% at 1550 nm under − 1 V bias. The GeSe/WS_2_/MoS_2_ heterostructure demonstrated relatively fast response times in the millisecond range under self-powered operation, whereas the rGO/Si_3_N_4_/GaAs device showed high responsivity (277 mA/W) and excellent detectivity at zero bias.

Compared with previously reported self-powered photodetectors, the Si_3_N_4_-2 device presented in this work achieved competitive broadband photodetection performance at 351, 500, and 1000 nm with responsivity values of 1.59, 4.65, and 8.73 mA/W, respectively, and detectivity values reaching 1.36 × 10^9^ Jones without any external bias. In addition, the device exhibited stable rise/fall response characteristics and appreciable EQE values, confirming the effectiveness of the Si_3_N_4_ interlayer in facilitating carrier separation and transport under self-powered operation. These results highlight the strong potential of the proposed Si_3_N_4_-based architecture for broadband, low-power, and self-powered optoelectronic applications.

**Table 1:** Comparison of broadband photodetection characteristics of previously reported devices with the Si_3_N_4_ photodetector fabricated in this study.

Interlayers	λ (nm)	*R* (mA/W)	D* (Jones)	EQE	τ_rise_/τ_fall_	Applied bias (V)	References
Ta_2_O_5_/ MWCNT	635	81.07	16.38 × 10^6^	15.5	80 ms/–	–	^63^
p-GaN/n-ZnO	358	0.68	-	–	–	0	^64^
GeSe/WS_2_/ MoS_2_	405	14	7.3 × 10^8^	4.1	2.4/5.2 ms	0	^65^
SnSe_2_/ PEDOT: PSS	1064	1.4 × 10^− 3^	2.8 × 10^8^	2.8 × 10^− 4^	1330 ms/-	0	^66^
TiO_2_/NiO	350	4.2 × 10^− 2^	1.1 × 10^9^	-	1.2/ 7.1 s	0	^67^
SiO_2_/Si_3_N_4_	1550	880	2.26 × 10^10^	70.68	–	− 1	^68^
Si_3_N_4_/Al_2_O_3_/ ReS_2_	365	2470	4.17 × 10^9^		0.26 s/1.96 s	10	^69^
rGO/Si_3_N_4_/ GaAs	572.28	277	0.67 × 10^10^	–	–	0	^70^
Si_3_N_4_-2	351	1.59	2.49 × 10^8^	0.588	251/301 ms	0	This work
	500	4.65	7.28 × 10^8^	1.206	137/863 ms		
	1000	8.73	1.36 × 10^9^	1.014	105/1208 ms		

The electrical, photoresponse, and spectral features of Si_3_N_4_/n-Si hybrid photodiodes were thoroughly analyzed, revealing that interfacial layer engineering is crucial in determining device performance. The I–V characteristics revealed that the ideality factor reduces with increasing illumination intensity, although the barrier height varies structurally, indicating increased carrier transport and interface quality under illumination.

The photoresponse analysis showed that photosensitivity (K), responsivity (R), and specific detectivity (D*) are strongly influenced by the Si_3_N_4_ interfacial configuration. The Si_3_N_4_-3/n-Si structure showed the maximum photosensitivity, while the Si_3_N_4_-2/n-Si device had the best overall optoelectronic performance, including strong responsivity, enhanced detectivity, and low noise equivalent power (NEP). The linear behavior of the log(I_ph_)-log(P) characteristics supported a photoconduction mechanism driven by trap states within the mobility gap. The Si_3_N_4_ interfacial layer enhances light absorption and carrier collection efficiency, leading to a wider photoresponse across the UV-Vis-NIR spectrum and higher external quantum efficiency (EQE). The generation of photogenerated carriers is a central mechanism governing the optoelectronic response of the fabricated Si_3_N_4_/n-Si photodiodes. Under illumination, electron–hole pairs are produced within the hybrid structure and subsequently separated at the Si₃N₄/Si interface, thereby reducing recombination losses and enhancing charge transport efficiency. This process is consistent with prior reports on carrier generation and separation in advanced semiconductor heterostructures, where defect states and interfacial engineering play a decisive role in modulating carrier dynamics^71^. By situating our findings within this established framework, the present study underscores the importance of interface-controlled photocarrier generation in achieving high-performance broadband photodetection. Overall, the findings indicate that managing interfacial layer thickness and properties is crucial for striking a balance between carrier generation, transport, and recombination processes.

## Conclusion

The study successfully fabricated Si_3_N_4_/n-Si hybrid photodiodes with various interfacial layer designs and systematically analyzed how interface engineering affects device performance. The results demonstrate that introducing a Si_3_N_4_ interfacial layer improves the optoelectronic characteristics of the photodiodes by enhancing carrier transport, reducing recombination losses, and modifying the barrier properties at the metal–semiconductor junction. Among the fabricated devices, the Si_3_N_4_-2/n-Si photodiode exhibited comparatively balanced performance in terms of responsivity, specific detectivity, and noise equivalent power under the investigated conditions. In contrast, although the Si_3_N_4_-3/n-Si device showed higher photosensitivity, its performance was partially limited by increased series resistance, highlighting the importance of optimizing interfacial layer thickness and composition. This behavior may be associated with residual impurities and oxygen-related interfacial states in Si_3_N_4_-2, which can facilitate carrier transport through modification of the barrier characteristics, whereas the relatively higher purity of Si_3_N_4_-3 may contribute to increased series resistance. Furthermore, the devices demonstrated broadband spectral response and enhanced external quantum efficiency across the UV–Vis–NIR region, indicating their potential for broadband photodetection applications. These findings suggest that Si_3_N_4_ interface engineering represents a promising strategy for improving silicon-based photodiode performance. Future work should include fabrication and statistical evaluation of a larger number of devices to further verify reproducibility and optimize device performance for CMOS-compatible optoelectronic applications.

## Supplementary Information

Below is the link to the electronic supplementary material.

## Data Availability

The datasets generated and/or analyzed during the current study are available from the corresponding author on reasonable request.
