# Supplementary material for: Interface-controlled broadband photodetection in Si3N4/n-Si hybrid devices
Source: Sci Rep. 2026 Jun 16;16:27627. doi: 10.1038/s41598-026-57951-y (PMC13538364; doi:10.1038/s41598-026-57951-y)
Supplement: Supplementary file 2 [file 41598_2026_57951_MOESM2_ESM.docx]

Highlights:

- Fabrication of Al/Si₃N₄/n-Si hybrid photodiodes using commercial and synthesized Si₃N₄ interfacial layers.
- Interface engineering significantly enhances carrier transport, reduces recombination, and improves junction quality.
- Optimized Si₃N₄-2/n-Si device exhibits superior responsivity, detectivity, and low noise equivalent power.
- Broadband photodetection achieved across the UV–Vis–NIR region with improved external quantum efficiency.
