# Supplementary material for: Interface-controlled broadband photodetection in Si3N4/n-Si hybrid devices
Source: Sci Rep. 2026 Jun 16;16:27627. doi: 10.1038/s41598-026-57951-y (PMC13538364; doi:10.1038/s41598-026-57951-y)
Supplement: Supplementary file 3 [file 41598_2026_57951_MOESM1_ESM.docx]

Graphical Abstract
